# Poloxamer sols endowed with in-situ gelability and mucoadhesion by adding hypromellose and hyaluronan for prolonging corneal retention and drug delivery

**DOI:** 10.1080/10717544.2022.2158964

**Published:** 2023-01-01

**Authors:** Ling-Chun Chen, Shyr-Yi Lin, Wei-Jie Cheng, Ming-Thau Sheu, Chi-Yun Chung, Chen-Hsuan Hsu, Hong-Liang Lin

**Affiliations:** a Department of Biotechnology and Pharmaceutical Technology, Yuanpei University of Medical Technology, Hsinchu, Taiwan, ROC; b Division of Gastroenterology, Department of Internal Medicine, Wan Fang Hospital, Taipei Medical University, Taipei, Taiwan, ROC; c Department of General Medicine, School of Medicine, College of Medicine, Taipei Medical University, Taipei, Taiwan, ROC; d Center for Drug Evaluation, Taipei, Taiwan, ROC; e School of Pharmacy, College of Pharmacy, Taipei Medical University, Taipei, Taiwan, ROC; f School of Pharmacy, College of Pharmacy, Kaohsiung Medical University, Kaohsiung, Taiwan, ROC

**Keywords:** Thermosensitive in situ Hydrogel, Poloxamer 407, HPMC, Hyaluronic acid, Hydroxypropyl β-cyclodextrin, Testosterone

## Abstract

The purpose of this study was to develop poloxamer (P407)-based in-situ thermogellable hydrogels with reducing concentration of P407 by adding hypromellose (HPMC) and with enhancing mucoadhesion of resulting hydrogels by adding hyaluronic acid (HA) for prolonging ocular delivery of hydroxypropyl-β-cyclodextrin (HPβCD)-solubilized testosterone (TES). Results demonstrated that 0.5% TES solution was successfully solubilized with adding 10% HPβCD. Non-gellable 13% P407 sol became in-situ gellable with adding 2.0-2.5% HPMC and mucoadhesibility was further imporved with adding 0.3% HA-L (low MW) or HA-H (high MW). Optimized 0.5% HPβCD-solubilized TES P407-based thermogellable hydrogels with enhancement of mucoadhesion for prolonging ocular delivery comprised 13% P407, 2.5% HPMC, and 0.3% HA-L or HA-H. Furthermore, rheological measurements under simulated eye blinking confirmed that non-thixotropic properties of optimized hydrogels could be spreaded evenly and retain a greater amount of drug-loaded hydrogels on the ocular surface for a longer period to prolong drug delivery. Compared with conventional eye drops, the prolonged residence time of optimized hydrogels from ex vivo and in vivo studies were observed, indicating relationships between rheological properties and in vivo performances. It was concluded that P407-based thermosensitive hydrogels with reducing concentration of P407 and enhancing mucoadhesion was successfully formulated by adding 2.5% HPMC and 0.3% HA in 13% P407 for potentially accomplishing effective clinical treatment of DED.

## 1. Introduction

Most patients with Dry eye disease (DED) experience chronic eye irritation with mild to moderate symptoms, including dryness, eye redness, general irritation. Thus, any causative factor of the disease should be treated using different mechanisms, such as improving tear production, reducing tear evaporation, slowing tear resorption, and alleviating ocular surface inflammation (Akpek et al., 2019). In a previous study, 86% patients with DED were diagnosed as having meibomian gland dysfunction (Lemp et al., 2012). Since testosterone might increase meibomian gland secretion and play a crucial role in secreting an oil layer and reducing water evaporation from the tear film. Therefore, testosterone may be a treatment option for most patients with DED (Sullivan et al., 2002; Supalaset et al., 2019). However, testosterone can cause severe systemic side effects and the US FDA issued a warning that testosterone might increase the risks of heart attack and stroke (Seftel, 2015; Ohlander et al., 2016). Further based on a systematic review of clinical studies of applying androgen in the treatment of DED, the administration of testosterone through an ocular drug delivery system (ODDS) can be a favorable option for DED with minimal systemic side effects (Wang & Deng, 2020).

Given that DED affects the precorneal and anterior area, hydrogel can be used as a drug delivery system (DDS), which enhances viscosity and increases both precorneal residence time and transcorneal permeability. In-situ forming hydrogel is a potential dosage form for the ODDS, which is in the aqueous phase during administration to the ocular and then undergoes gelation to form viscoelastic hydrogel upon contact. Further, this gel-like structure also provides the advantages of enhanced bioavailability, minimized systemic absorption, and decreased dosage frequency (Wu et al., 2019). Thermosensitive, pH-sensitive, and ion-activated in-situ hydrogels are the three most thoroughly investigated hydrogels. Thermosensitive in-situ hydrogels are safer than pH-sensitive and ion-activated in-situ hydrogels concerning the risk of irritation and damage to eyes and conjunctival cells (Wei et al., 2020). Accordingly, thermosensitive in-situ hydrogel was selected as the dosage form for the ODDS of testosterone.

Poloxamer 407 (P407) was chosen as the thermosensitive polymer in the ODDS in this study. An appropriate concentration of P407 can covert solution phase into the gel phase under body temperature. P407 solution also exhibits mucomimetic properties and optical clarity and thus can be used as a tear substitute (Bourlais et al., 1998). Moreover, P407 solution cause little damage to the mouse or rabbit cornea, indicating the excellent safety of P407 solution (Furrer et al., 2000). However, although the administration of 20% P407 formulation four times a day for 3 days was safe for the animal cornea, the safety of the long-term administration of this dosage remains unclear. Therefore, to reduce the total amount of P407 used in formulations and ensure the rheological behavior and in-situ gelling effect, studies have proposed the use of a combination of P407 with a viscosity enhancer (Almeida et al., 2013; Escobar-Chávez et al., 2006).

Hyaluronic acid (HA) can relieve irritation, moisturize the ocular surface, and overcome the sodium hyaluronate deficiency in the tear film, thus alleviating DED symptoms. Hence, HA is the main ingredient in artificial tear formulations currently used for treating DED and Sjögren’s syndrome (Yang et al., 2021). Moreover, the mucoadhesive property and high viscosity of HA (Sudha & Rose, 2014) prevents the rapid washout of the drug by tears, thus prolonging the residence of drug and increasing ocular drug availability (Salwowska et al., 2016). Besides, the high molecular weight (MW) of HA inhibits inflammation by suppressing the production of inflammatory cytokines (Ruppert et al., 2014), which is beneficial for DED (Yamaguchi, 2018). The advantages of the cellulose derivatives of hydroxypropyl methylcellulose (HPMC) include safety, biocompatibility, and mucoadhesive property, which make them suitable for use as ODDS. Moreover, the mucoadhesive property of HPMC enables long contact time, allowing the drug to easily permeate the eye tissue (Tundisi et al., 2021). Therefore, we hypothesize that the addition of HPMC and HA would convert nongellable P407 aqueous solution into an in-situ gellable hydrogel with enhanced mucoadhesive characteristics. Accordingly, we developed P407-based thermosensitive in-situ hydrogels combined with HPMC and HA to reduce the desired concentration of P407 required for maintaining the formation of in-situ hydrogels with an enhanced mucoadhesive property to increase residence time for the continuing ocular delivery of solubilized testosterone in this study.

## 2. Materials and methods

### 2.1. Materials

Testosterone and fluorescein sodium salt were purchased from Sigma-Aldrich Co. Ltd. (St. Louis, MO, USA). P407 [poly(ethylene glycol)_95-105_-block-poly(propylene glycol)_54-60_-block-poly(ethylene glycol)_95-105_; average MW: 9840–16,400] was procured from BASF Co. Ltd. (Ludwigshafen, Germany). α-Cyclodextrin (αCD), β-Cyclodextrin (βCD), Hydroxypropyl β-Cyclodextrin (HPβCD), and γ-Cyclodextrin (γCD) were purchased from Nakarai Chemicals Co. Ltd (Kyoto, Japan). HA (HA-L, MW: 700–900 K; HA-H, MW: 2000–2200 K) was purchased from Biotech Co. Ltd (Shanghai, China). HPMC 606 [hypromellose 606, viscosity (2% w/w aqueous solution at 20 °C): 4.8–7.2 cp; substitution type: 2910; methoxy content: 28.0%–30.0%; hydroxypropoxy content: 7.0%–12.0%] was purchased from Shin-Etsu Chemical Co. Ltd (Tokyo, Japan). New Zealand albino rabbits (weight: 2.0–3.0 kg) were supplied by Livestock Research Institute (Tainan, Taiwan).

### 2.2. Measurement of testosterone solubility

Saturation solubility was examined using the standard shake-flask method reported previously (Balguri et al., 2016). An excess amount of testosterone was added to centrifuge tubes containing 6 mL of distilled water with 5% (w/v) solubilizers, namely αCD, βCD, γCD, HPβCD, and P407. To ensure uniform mixing, the samples were stirred at 100 rpm for 24 h at 25 °C. After 24 h, the samples were centrifuged at 8000 rpm for 10 min, and the supernatant was analyzed using the high-performance liquid chromatography (HPLC) with ultraviolet detection (HPLC-UV) method.

Testosterone was analyzed using an HPLC-UV system (Hitachi Co., Ltd., Tokyo, Japan) with a reverse-phase LiChrospher 100 RP-18 column (5 μm, 125 mm × 4 mm, Merck) and a C_18_ guard column. The mobile phase was acetonitrile:water in a 60:40 (v/v) ratio, pumped at 1.0 mL/min. The injection volume was 5 μL, and absorbance was monitored at 241 nm. Under these conditions, testosterone retention time was approximately 5 min. The calibration curve was plotted in the concentration range of 0.2–20 μg/mL. The precision and accuracy of the HPLC method were validated before implementation.

### 2.3. Optimization of poloxamer-based thermosensitive hydrogels

#### 2.3.1. Preparation of P407-based in situ hydrogels

Thermosensitive in situ hydrogels were prepared using a physical mixing method as follows: Briefly, appropriate amounts of P407, HPMC, and HA (HA-L and HA-H) were weighed and subsequently added in distilled water in a predetermined composition (w/w). The solution was mixed through mechanical vortexing and continuous agitation at 500 rpm in a 4 °C refrigerator until a transparent solution was obtained. For the formulation of the drug to be loaded in in situ hydrogels, 0.5% testosterone and 10% (w/w) HPβCD with distilled water were used as the solution of in situ hydrogels. The pH of the system was adjusted to 7.4 ± 0.1 by using 1 M NaOH solution.

#### 2.3.2. Solution–gel phase transition temperature

The solution–gel (sol–gel) transition temperatures of different in situ hydrogels (as displayed in Table 1) were determined using the vial inversion method (Wei et al., 2020). Briefly, 0.5 mL of the tested formulation was added to a vial placed in a water bath. The temperature of the water bath was increased at 2 °C/step (4 °C, 20 °C–40 °C). The formulation was equilibrated for 5 min and then visually observed for gelation by inverting the vial at a setting temperature. The sol–gel transition temperature was determined when the formulation was fixed to the bottom of the vial within 10 s in the gel state. If the formulation maintained the state of the solution in the range of the entire setting temperature, the formulation was considered to not exert a gelation effect on the ocular surface.

**Table 1.** Effect of the HPMC and HA content on the status of 13% P407 gel at 4, 25 and 34 °C.

Acronym	P407 (%)	HPMC (%)	HA (%) H:2000KDa L:7000KDa	Status at different temperatures		
				4 °C	25 °C	34 °C
P_13_	13	–	–	Solution	Solution	Solution
P_13_HA-L_0.1_	13	–	0.1 HA-L	Solution	Solution	Solution
P_13_HA-H_0.1_	13	–	0.1 HA-H	Solution	Solution	Solution
P_13_HA-L_0.3_	13	–	0.3 HA-L	Solution	Solution	Solution
P_13_HA-H_0.3_	13	–	0.3 HA-H	Solution	Solution	Solution
P_13_H_2.0_	13	2.0	–	Solution	Solution	Gel
P_13_H_2.0_HA-L_0.1_	13	2.0	0.1 HA-L	Solution	Solution	Gel
P_13_H_2.0_HA-H_0.1_	13	2.0	0.1 HA-H	Solution	Solution	Gel
P_13_H_2.0_HA-L_0.3_	13	2.0	0.3 HA-L	Solution	Solution	Solution
P_13_H_2.0_HA-H_0.3_	13	2.0	0.3 HA-H	Solution	Solution	Solution
P_13_H_2.5_	13	2.5	–	Solution	Solution	Gel
P_13_H_2.5_HA-L_0.1_	13	2.5	0.1 HA-L	Solution	Solution	Gel
P_13_H_2.5_HA-H_0.1_	13	2.5	0.1 HA-H	Solution	Solution	Gel
P_13_H_2.5_HA-L_0.3_	13	2.5	0.3 HA-L	Solution	Solution	Gel
P_13_H_2.5_HA-H_0.3_	13	2.5	0.3 HA-H	Solution	Solution	Gel

H: symbolized HPMC, the subscript of C symbolized the concentration of HPMC (w/w).

HA-L: symbolized low molecular weight of HA (MW:700–900K), the subscript of HA-L symbolized its concentration (w/w).

HA-H: symbolized high molecular weight of HA (MW:2000–2200K), the subscript of HA-H symbolized its concentration (w/w).

#### 2.3.3. Physical characterization through dynamic light scattering

The particle diameter and zeta potential of the micelle formation of P407-based in situ hydrogels were determined through dynamic light scattering (DLS) by using a zeta potential and particle size analyzer (ELSZ-2000, Otsuka Electronics Co., Ltd., Osaka, Japan). All formulations were diluted in deionized water at a ratio of 1:100 and maintained at 25 °C until zeta potential measurement. Meanwhile, the samples were diluted in deionized water at a ratio of 1:200 and maintained at 25 °C and 34 °C until particle size measurement. Data were analyzed using ELSZ-2000 software (version 7.11).

### 2.4. Preparation and rheological study of optimal testosterone-loaded poloxamer-based thermosensitive hydrogels

The composition of optimal testosterone-loaded P407-based in situ hydrogels is listed in Table 2 and prepared following the aforementioned procedure except that 0.5% (w/w) testosterone was solubilized in 10% (w/w) HPβCD in double deionized water (DD water) before dissolving the rest of components in the solution. Drug solution prepared by dissolving 0.5% (w/w) testosterone with 10% (w/w) HPβCD in DD water was used as control. The pH of the so-obtained solution was adjusted to 7.4 ± 0.1 by using 1 M NaOH solution. To simulate physiological situation of instilling the in situ hydrogels into the conjunctival sac, we performed a rheological analysis after the dilution of the in situ hydrogels with simulated tear fluid (STF) at a 40:7 v/v ratio. This ratio of dilution was used because the volume of the lacrimal fluid in the eye is 7 μL, and the drop volume of commercial eye solutions is approximately 40 μL (Destruel et al., 2020). STF was prepared as follows: 67 mg of sodium chloride was dissolved in 200 mg of sodium bicarbonate. Then, 8 mg of calcium chloride dihydrate was dissolved in 100 mL of water for injection, and the pH of the resulting solution was adjusted to 7.4 ± 0.1 by using 5 M NaOH. The rheological properties of all in situ hydrogels were examined using the Anton Paar MCR302 Rheometer (Anton Paar GmbH, Graz, Austria). Cone and plate geometry with a diameter of 50 mm, a cone angle of 1.0°, and a truncation gap of 0.097 mm was used for all rheological analyses. Four types of rheological properties were characterized.

**Table 2.** The status of optimal testosterone-loaded (0.5%) P407-based (13%) measured by vial inversion method at 4, 25 and 34 °C and sol-gel transition temperature.

Acronym	P407 (%)	HPMC (%)	HA (%) H:2000KDa L:7000KDa	Status at different temperatures			Sol-gel transition temperature (T_sol-gel_, °C)
				4 °C	25 °C	34 °C	
TES/P_13_H_2.5_	13	2.5	–	Sol	Sol	Gel	26.72 ± 1.38
TES/P_13_H_2.5_HA-L_0.3_	13	2.5	0.3 HA-L	Sol	Sol	Gel	24.10 ± 0.52
TES/P_13_H_2.5_HA-H_0.3_	13	2.5	0.3 HA-H	Sol	Sol	Gel	23.53 ± 0.58
TES/P_13_H_2.5_-STF	13	2.5	0.3 HA-H	Sol	Sol	Sol	28.81 ± 0.63
TES/P_13_H_2.5_HA-L_0.3_-STF	13	2.5	0.3 HA-L	Sol	Sol	Sol	26.70 ± 1.32
TES/P_13_H_2.5_HA-H_0.3_-STF	13	2.5	0.3 HA-H	Sol	Sol	Sol	26.33 ± 1.79

H: symbolized HPMC, the subscript of H symbolized the concentration of HPMC (w/w); STF: simulated tear fluid.

HA-L: symbolized low molecular weight of HA (MW:700–900K), the subscript of HA-L symbolized its concentration (w/w).

HA-H: symbolized high molecular weight of HA (MW:2000–2200K), the subscript of HA-H symbolized its concentration (w/w).

#### 2.4.1. Temperature ramp analysis

After equilibrium at 20 °C for 3 min, the temperature was gradually increased from 20 °C to 40 °C at a heating rate of 0.5 °C/min, whereas the sample was sheared at a fixed rate of 1.0 s ^− 1^. Shear stress and apparent viscosity were determined as a function of temperature.

#### 2.4.2. Steady shear deformation for pseudoplastic flow behavior

The shear rate was changed from 0.01 to 2000s ^−1^, and stress was measured at a precorneal temperature of 34 °C. Viscosity was then calculated.

#### 2.4.3. Oscillatory deformation (amplitude sweep)

The in situ hydrogels were loaded and left to equilibrate in the rheometer for 3 min at a precorneal temperature of 34 °C. Then, amplitude sweep was performed. The strain amplitude varied from 0.01% to 200% at a fixed frequency of 10 rad/s. The elastic (G′) and viscous (G′′) moduli of the in situ hydrogels were determined as a function of strain amplitude.

#### 2.4.4. Oscillatory deformation (frequency sweep)

The frequency was changed from 0.1 to 100 rad/s. The G′ and G′′, respectively, were determined as a function of the frequency of oscillations at a constant amplitude of deformation (Shear strain ≈ 0.1%) and a precorneal temperature of 34 °C.

### 2.5. Gel resistance under simulated eye blinking

#### 2.5.1. Rotational measurements

This measurement was designed to simulate the behavior of in situ hydrogels under eye blinking after instillation. Hydrogel formulations were alternatively subjected to a high destructive shear rate of 5000s ^− 1^ for 1 s, immediately followed by measurements at a low destructive shear rate of 0.03 s ^− 1^ for 1 min. The high destructive shear rate of 5000s ^− 1^ was selected to simulate the physiological eye blinking shear rate that was reported to range from 3000 to 40,000 s ^− 1^ (Destruel et al., 2020). A low shear rate of 0.03 s ^− 1^ was used to mimic the environment of an interblinking eye and examine viscosity behavior. This measurement was conducted ten times in a row to simulate 10 eye blinks. The physiological interblinking period is approximately 5–7 s, but a 1-min interval was required to achieve sufficient precision of the measurement. This measurement was also conducted after dilution with STF.

#### 2.5.2. Oscillatory measurement

The same measurement was performed by replacing the low shear rate measurement phase by oscillatory measurement to determine the elastic behavior of in situ hydrogels under eye blinking. Thus, hydrogels were assessed alternatively at a high shear rate of 5000s ^− 1^ for 1 s, immediately followed by nondestructive oscillatory measurements at a frequency of 1 Hz and an amplitude of 0.1% for 1 min. By contrast, oscillatory measurements were selected to examine the elastic behavior under simulated eye blinks. This test was performed 10 times in a row to simulate 10 eye blinks. This measurement was also conducted after dilution with STF.

### 2.6. Ex vivo transcorneal study

Dissection of the rabbit corneas was performed by Riddle Technologies Co. LTD (Hsinchu, Taiwan). The whole cornea with the sclera around was removed and stored in sterilized normal saline at 4 °C to transport in 2 to 3 hours. The experiment was immediately conducted after receiving the corneas. The ex vivo transcorneal permeation study of in situ hydrogels was conducted using a Franz diffusion cell (Fathalla et al., 2016; Tan et al., 2019). The excised cornea was clamped between the donor and receptor compartment of a Franz diffusion cell in such a manner that the epithelial side was placed toward the donor compartment. The corneal area for diffusion was 0.785 cm^2^. A volume of 1.0 mL of each in situ hydrogel containing 5 mg of testosterone was added to the donor compartment, which was covered with parafilm to prevent the evaporation of the formulation. Because of the unavailability of a commercial eye drop containing testosterone for the treatment of DED, 0.5% testosterone with 10% HPβCD in distilled water was prepared as control (drug solution). The receptor chamber (5 mL volume) was filled with phosphate-buffered saline (pH 7.4) with constant magnetic bar stirring (400 rpm) at 34 °C ± 1 °C. At planned time intervals (0.15, 0.5, 1.0, 2.0, 4.0, 6.0, 8.0, 12.0 and 24.0 h), 0.5 mL aliquots of the receptor solution were withdrawn for measurement, and an equal volume of diffusion medium was replaced. The samples were analyzed through HPLC, and data are reported as the mean ± standard deviation (*n* = 3).

### 2.7. Ex vivo precorneal residence study

The residence time of different formulations on the ocular surface was investigated as described in the literature (Cave, Cook, Connon, & Khutoryanskiy, 2012; Al Khateb et al., 2016; Cook et al., 2015). Briefly, the corneas were placed on the curved device, which was fixed on the glass slide to mimic the curvature of the human eye. Then, 200 μL of the formulation with 1 mg/mL of fluorescein sodium was instilled on the cornea in an incubator at 34 °C. After equilibrating for 3 min to ensure the gelation of in situ hydrogels, the whole slide was moved to the device tilted at 30° in the same incubator. The samples were subjected to many continuous washes with 200 μL of STF to wash off testing substrates from the surface of the cornea. To imitate eye blinking, each wash was considered as one blink; in this period of time, 10 washes were conducted. The photos were obtained after each wash by using the camera of the cellphone with weak UV light from an ultraviolet torch. The cellphone with the flat structure of camera lens was placed on a shelf at a fixed height to confirm that the area of the same cornea among different photos was duplicate. From these images, fluorescence samples remaining on the cornea were analyzed using ImageJ (Version 1.8.0). The threshold function was used at a saturation of 100 and a brightness of 100 to analyze all photos, and the coverage was measured using the ‘Analyze Particles’ function built in ImageJ (). Similar steps were conducted on glass slides without the corneal tissue to examine the residence time of formulations directly. Data are reported as the mean ± SD (*n* = 3).

### 2.8. In vivo precorneal residence study

Animal studies were approved by the Animal Ethical Committee of Kaohsiung Medical University and complied with the Principles of Laboratory Animal Care. The ethical committee approval number for animal studies is IACUC 110046. New Zealand albino rabbits (weight: 2.0 to 3.0 kg) were purchased from Livestock Research Institute (Tainan, Taiwan). The in vivo precorneal residence study was conducted as described previously (Al Khateb et al., 2016). Rabbits were placed in standard cages and provided ad libitum access to food and water. The rabbits were placed in restraining boxes, and we ensured that they could blink without any restrictions. The formulations containing fluorescein sodium (70 mL at 1 mg/mL) were instilled on the rabbit’s ocular surface. The tear fluid was carefully collected from the lower part of the rabbit’s eye surface with cotton buds at predominant time intervals (0, 5, 10, 15, 20, 25, 30, 40, 50 and 60 min). The cotton buds were placed in 1 mL of 90% methanol solution for 24 h to extract testosterone. After centrifugation at 8000 rpm for 10 min, the extract was analyzed through HPLC. Photos were obtained using the camera of the cellphone, which was fixed on the tripod to ensure that the area of the same cornea among different photos was duplicated. Weak UV light from an ultraviolet torch was used to improve the detection of fluorescein sodium in formulations. Similar to the ex vivo precorneal residence study, all photos were analyzed and the subsequent coverage was measured using the ‘Analyze Particles’ function built in ImageJ (Version 1.8.0). Data are reported as the mean ± SD (*n* = 4).

### 2.9. Statistical analysis

All text data are plotted as graphs and were analyzed using PRISM (v6.0 GraphPad Inc.). All mean values are expressed in SD. One-way and two-way ANOVA was used for data analysis within and between groups, respectively. ‘*’ represents a significant difference at *P* < .05). ‘**’ represents a significant difference at *P* < .01, ‘***’ represents a significant difference at *P* < .005, ‘****’ represents a significant difference at *P* < .001.

## 3. Results and discussion

### 3.1. Measurement of testosterone solubility

To ensure that lipophilic testosterone can be loaded in the hydrophilic in situ gelling system and enhance ocular bioavailability, we examined the solubility of testosterone in different types of solubilizers and the results are presented in Figure S1. We first evaluated the solubility of P407, which is a thermosensitive polymer and surfactant. The solubility of testosterone did not increase in 5% P407. A previous study reported that a 0.5% testosterone eye drop might be beneficial to patients with DED (Dawson, 2015). We noted that 0.5% testosterone did not solubilize in the presence of 13% P407, indicating the necessity of another solubilizer for the formulation.

**Figure 1.**
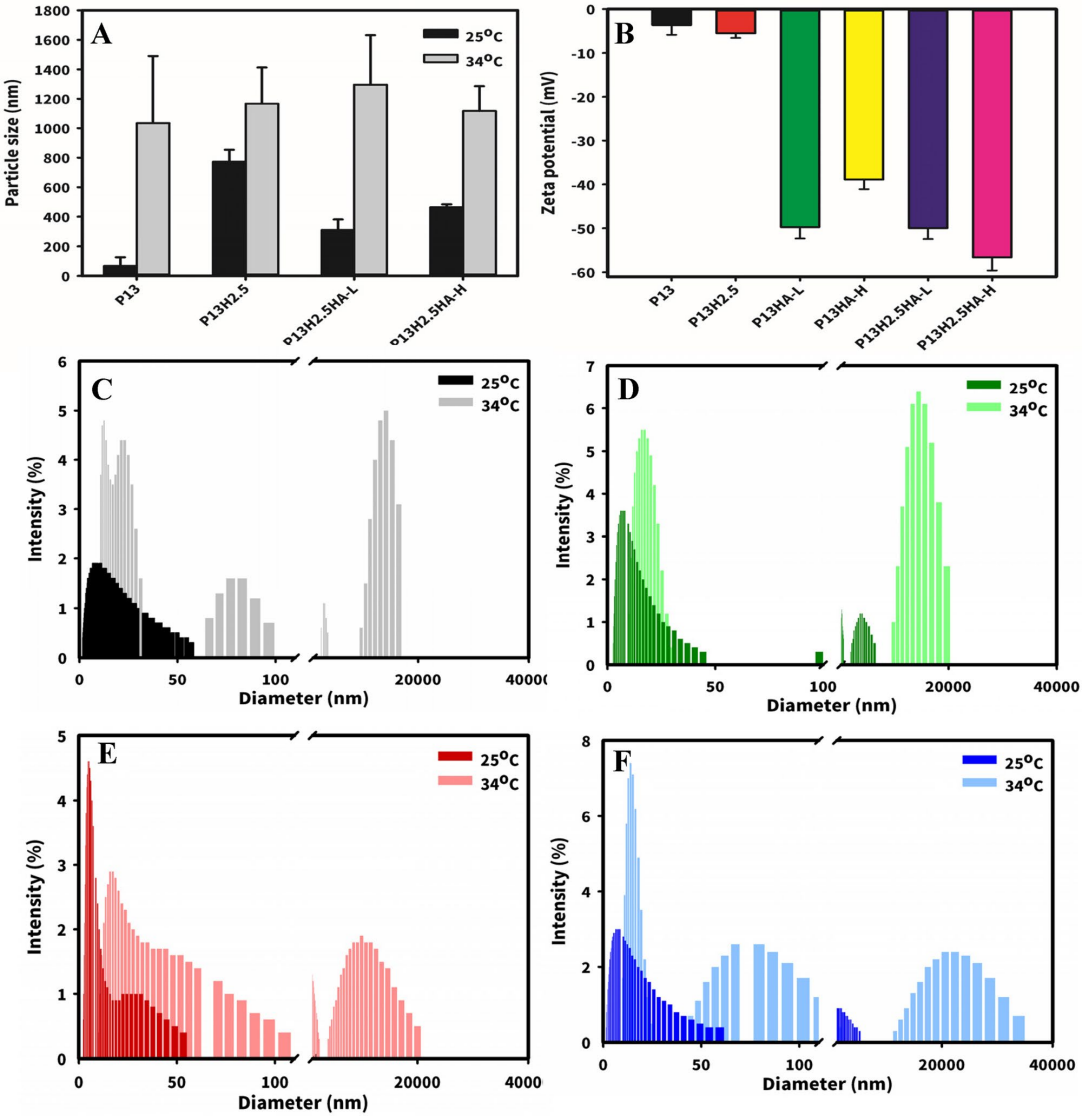
Effect of the addition of HPMC and HA on the mean particle size (A) and zeta potential (B) of 13% P407 gels (P_13_, P_13_H_2.5_, P_13_HA-L_0.3_, P_13_HA-H_0.3_, P_13_H_2.5_HA-L_0.3_, and P_13_H_2.5_HA-H_0.3_), and the particle size distribution of P_13_ (C), P_13_H_2.5_ (D) P_13_H_2.5_HA-L_0.3_ (E) and P_13_H_2.5_HA-H_0.3_ (F) at 25 °C and 34 °C (* represents a significant difference at *P* < .05, ** represents a significant difference at *P* < .01, *** represents a significant difference at *P* < .005 compared with the mean particle size of 13% P407 gels).

CDs are cyclic oligosaccharides composed of (α-1,4)-linked D-glucopyranose units. CDs solubilize hydrophobic drugs by forming a water-soluble inclusion complex of lipophilic insoluble drugs and take up the lipophilic domain of the drugs into the central cavity. CDs can be used as a permeation enhancer in drug delivery systems because they increase the concentration of the dissolved drug at the membrane surface and deliver it to the surface of the corneal barrier where they undergo partition into the eye (Jansook et al., 2018). Among α-CD, β-CD, γ-CD, and HPβCD examined in this study, only HPβCD effectively improved the water solubility of testosterone. Thus, we used HPβCD as a solubilizer in this system. We employed 10% HPβCD to solubilize 0.5% testosterone. A study reported that 12.5% HPβCD was well tolerated by the rabbit eye and did not damage the corneal epithelium (Jansen et al., 1990).

### 3.2. Optimization of P407-based thermosensitive hydrogels

#### 3.2.1. Sol–gel phase transition study

We investigated the effect of the different concentrations of P407 (11%–22%) on the sol–gel phase transition temperature for the resulting P407 solution, and the results are presented in Figure S5A. We observed that 11% and 13% P407 in situ gels did not exhibit gelation at body temperature. The P407 concentration was negatively correlated with the sol–gel gelation temperature. When the P407 concentration ranged from 17% to 22%, the gelation temperature, which decreased with an increase in the P407 concentration, was <25 °C. This finding indicated that the formulations prepared using 17%–22% P407 were converted into gel at room temperature, resulting in an inaccurate dosage and blurred vision at the time of administration. When the P407 concentration was 15%, the gelation temperature was 28 °C; this concentration maintained the solution state at the beginning of drug administration at room temperature, after which the solution transformed into the gel phase on the ocular surface. Thus, when a single type of polymer is used, 15% P407 can be more suitable for preparing in situ gels for ocular drug delivery.

Because of the surfactant property of P407, a high P407 concentration may increase the risk of corneal endothelial damage (Wei et al., 2020). Because of the chronic nature of the disease, patients with DED may require long-term therapy, which might result in the increased accumulation of P407. Thus, reducing the P407 concentration can prevent potential irritation to the eyes. Thus, for this purpose, the use of viscosity enhancers with mucoadhesive property, such as HPMC and HA, can be beneficial (Shastri et al., 2010; Jung et al., 2017). We observed that 13% P407 did not transfer into the gel state by itself at an appropriate temperature. Thus, we examined the effect of the addition of HPMC (0.5%–4.0%) on the gelation ability of 13% P407 solution, and the results are presented in Figure S5B. The concentration of HPMC in 13% P407 gels was negatively correlated with the sol–gel phase transition temperature. We noted that 13% P407 solution could be converted into the gel state at a precorneal temperature of 34 °C in the presence of 2.0% HPMC but at 26 °C in the presence of 4.0% HPMC. This finding indicated that the addition of an adequate HPMC concentration ensured gelation in the presence of a low P407 concentration. Furthermore, we investigated the effect of the addition of HA-H and HA-L on 13% P407 in situ gels, and the results are illustrated in Figure S5C. We did not observe the gelation of 13% P407 solution in the presence of 0.1%–0.5% HA-L. The same results were noted for HA-H. These findings indicate that HA is not suitable for the gelation of P407 solution.

We determined the sol–gel phase transition temperature for 13% P407 in situ gels (P_13_) containing both HPMC (H) and HA by using the vial inversion method, and the tested formulations are listed in Table 1. No sol–gel phase transition was noted for formulations containing P_13_ only and P_13_ containing HA-L and HA-H at 0.1% and 0.3% (P_13_HA-L_0.1,0.3_ and P_13_HA-H_0.1,0.3_), respectively. In the presence of 2.0% or 2.5% HPMC, P_13_ solution (P_13_H_2.0_) could be converted into a hydrogel at 34 °C. However, in the presence of 2.0% HPMC, P_13_H_2.0_ containing 0.1% HA-L or HA-H (P_13_H_2.0_HA-L_0.1_ or P_13_H_2.0_HA-H_0.1_) could be converted into the gel state at 34 °C. By contrast, in the presence of 2.5% HPMC, those formulations containing 0.1% and 0.3% HA-L and HA-H (P_13_H_2.5_HA-L_0.1_, P_13_H_2.5_HA-L_0.3_, P_13_H_2.5_HA-H_0.1_, and P_13_H_2.5_HA-H_0.3_), respectively, could be converted into the gel state at 34 °C. The results indicate that HA does not act as a gelling agent in P407 solution and might even reduce the gelation effect of formulations. By contrast, HPMC is a suitable option for enhancing the gelation effect on formulations in the presence of P_13_.

A study reported that 0.5% HPMC protected the ocular surface for a long term and effectively maintained corneal hydration (Toda et al., 1996). Application of 2% HPMC on the corneal surface during ocular surgery can improve the clinical outcomes of the tear film and ocular surface (He et al., 2017). Moreover, 0.3% HA protected against tear film instability or ocular surface dehydration and exhibited better efficacy in DED than 0.18% HA did when present in an artificial tear eye drop (You et al., 2018). In summary, considering the mucoadhesive property of HA, gelation effect of HPMC, and efficacy of both HPMC and HA for DED, we selected formulations containing 2.5% HPMC and 0.3% HA-L or HA-H (P_13_H_2.5_, P_13_H_2.5_HA-L_0.3_, and P_13_H_2.5_HA-H_0.3_) along with HPβCD-solubilized testosterone for further study (TES/P_13_H_2.5_, TES/P_13_H_2.5_HA-L_0.3_, and TES/P_13_H_2.5_HA-H_0.3_, respectively; Table 2).

#### 3.2.2. Physical characterization through DLS

Apart from observing the macroscopic characteristics of the formulation by performing an in vitro gelation study, we examined the microscopic mechanism underlying the micelle formation of P407 in situ gels through DLS. We investigated the effect of the addition of HPMC and HA in P_13_ on micellization. At 25 °C, the mean particle size of P_13_ was 67 ± 59 nm (Figure 1(A)), whereas those of P_13_H_2.5_, P_13_H_2.5_HA-L_0.3_, and P_13_H_2.5_HA-H_0.3_ were 772 ± 81, 310 ± 70, and 464 ± 19, respectively. Among them, the mean particle size of only P_13_H_2.5_ significantly differed from that of P_13_ (*P* < .05). Although the mean particle size of both P_13_H_2.5_HA-L_0.3_ and P_13_H_2.5_HA-H_0.3_ did not significantly differ from that of P_13_, their diameters were four and six times larger than that of P_13_. The temperature exerted a stronger effect on micellar aggregation. The mean particle size of all the formulations at 34 °C increased compared with their own particle size at 25 °C (Figure 1(A)). The micelles markedly clustered together in response to an increase in temperature to 34 °C, even leading to an increase of up to tens of thousands in the particle size of micelles in all the formulations.

P407 is a hydrophilic nonionic surfactant and a triblock copolymer composed of (PEO–PPO–PEO). Both its hydrophilic (PEO) and hydrophobic (PPO) properties lead to micelle formation when P407 is dissolved in aqueous solutions at a concentration above the critical micellar concentration (Russo & Villa, 2019). The gelation mechanism of P407 in situ gels was reported previously (Dumortier et al., 2006). Micellization occurs due to the dehydration of the PO domain of P407 to form a hydrophobic core of micellar structures along with an outer shell composed of the hydrated EO domain. With a rise in temperature, the effect of PO dehydration becomes stronger, resulting in increased micelle formation and thus rearrangement into a gel structure.

Except for the effect of temperature on the micellization of P407, the effect of a viscosity enhancer on micellar aggregation was deduced. P_13_ and P_13_H_2.5_ exhibited a near-neutral zeta potential (Figure 1(B)). By contrast, all the formulations containing HA (P_13_HA-L_0.3_, P_13_HA-H_0.3_, P_13_H_2.5_HA-L_0.3_, and P_13_H_2.5_HA-H_0.3_) displayed a negative zeta potential. These results might explain the previous inference that HA reduces the gelation effect of P407 gels for both P_13_HA-L_0.3_ and P_13_HA-H_0.3_. During the process of P407 micellar aggregation caused by an increase in temperature, the ionized carboxylic acid group (-COO^−^) of HA might apply a negative charge on the surface of micelles, thus increasing the distance between micelles with electrostatic repulsion and resulting in the decreased aggregation of micelles to be converted into a gel. However, the addition of 2.5% HPMC converted the nongellable P_13_HA-L_0.3_ and P_13_HA-H_0.3_ formulations into the in situ gellable P_13_H_2.5_HA-L_0.3_ and P_13_H_2.5_HA-H_0.3_ formulations, respectively. This can be attributed to enhanced viscosity by HPMC that weakened the negative electrostatic repulsion effect of HA, thus increasing the aggregation of micelles and leading to a gel state.

The mean particle size of only P_13_H_2.5_ significantly differed from that of P_13_ (*P* < .05). Although the mean particle size of both P_13_H_2.5_HA-L_0.3_ and P_13_H_2.5_HA-H_0.3_ did not significantly differ from that of P_13_ possibly due to electrostatic repulsion, their diameters were four and six times larger than that of P_13_, resulting in conversion to the gel state. Similar results are illustrated in the plot of particle size distribution (Figure 1©). At room temperature, the particle size of P407 micelles (P_13_) was approximately 12.9 ± 11.2 nm, which might represent the size of a single micelle. In the P_13_H_2.5_ group, we observed a similar peak representing the diameter of a single micelle (approximately 10 nm); however, the other peak (3860.0 ± 1171.3 nm) appeared to represent micellar aggregation. Similarly, we noted two micelle diameters in each P_13_H_2.5_HA-L_0.3_ and P_13_H_2.5_HA-H_0.3._

A previous study described the mechanism through which HPMC enhances the gelation effect (Ruel-Gariépy & Leroux, 2004). HPMC caused the dehydration of hydrophobic domains in HPMC molecular chains with an increase in temperature, leading to polymer–polymer interaction and eventually forming an infinite network structure. The attachment of micelles to the network structure can reduce the distance between micelles, thus facilitating their aggregation. When considering zeta potential and particle size together, we observed that in the formulations containing both HA and HPMC, although HA provided the negative charge on the micellar surface, an adequate concentration of HPMC still allowed micelles to attach to its framework and undergo aggregation.

#### 3.2.3. Viscoelastic measurement

**Figure 2.**
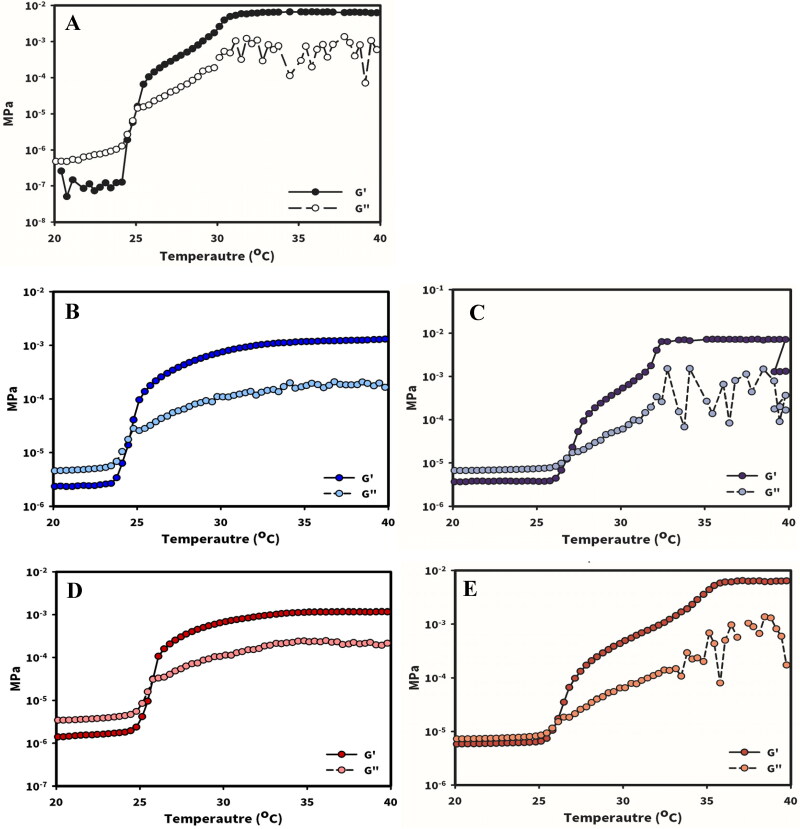
T_sol-gel_ temperature of P_13_H_2.5_ (A), P_13_H_2.5_HA-L_0.3_ (B), TES/P_13_H_2.5_HA-L_0.3_ (C), P_13_H_2.5_HA-H_0.3_ (D), and TES/P_13_H_2.5_HA-H_0.3_ (E) investigated by viscoelastic measurement.

Compared with the vial inversion method used to determine gelation temperature visually, oscillatory experiments were performed to examine the liquid or gel state of formulations. G′ represents the storage modulus, and G′′ represents the loss modulus. When the value of G′ was higher than that of G′′, the sample was observed to be in the gel state. Gelation temperature (T_sol-gel_) was defined as the crossover between the G′ and G′′ of the formulations (Figure 2). The T_sol-gel_ of P_13_H_2.5_ was 25.5 °C ± 0.6 °C. Even in the presence of a marked change in viscosity, the T_sol-gel_ of the formulations containing HA of both viscosity grades, P_13_H_2.5_HA-L_0.3_ and P_13_H_2.5_HA-H_0.3_ (25.2 °C ± 0.8 °C and 25.9 °C ± 1.2 °C, respectively) was similar to that of P_13_H_2.5_. In terms of different gelation properties, the addition of HA in P407 in situ gels had a stronger effect on viscosity than on T_sol-gel_. Moreover, the viscosity of all the formulations markedly increased above their T_sol-gel_ due to transformation from a solution state to a gel state. When HPβCD-solubilized testosterone was added in P_13_H_2.5_HA-L_0.3_ and P_13_H_2.5_HA-H_0.3_ (TES/P_13_H_2.5_HA-L_0.3_ and TES/P_13_H_2.5_HA-H_0.3_, respectively), their T_sol-gel_ were 26.7 °C ± 0.5 °C and 26.1 °C ± 0.4 °C, which are similar to those of the formulations without the drug. Although both G′ and G′′ for TES/P_13_H_2.5_HA-L_0.3_ and TES/P_13_H_2.5_HA-H_0.3_ slightly increased simultaneously before reaching their T_sol-gel_ with respect to that for P_13_H_2.5_HA-L_0.3_ and P_13_H_2.5_HA-H_0.3_, respectively, no difference in their T_sol-gel_ was noted. Overall, the findings indicated a nonsignificant effect of the addition of HPβCD-solubilized testosterone on the T_sol-gel_ of those formulations.

### 3.3. Rheological study of optimized P407-based thermosensitive hydrogels

The rheological properties of ophthalmic in situ gels have a considerable effect on accurate dosing, spreadability, retainability, and patient compliance. We examined the rheological characteristics of optimal in situ gels (P_13_H_2.5_, P_13_H_2.5_HA-L_0.3_, and P_13_H_2.5_HA-H_0.3_) to determine their applicability in in situ hydrogels as eye drops. Because the tear volume in normal is approximately 7 μL and a drop of a typical commercial eye drop solution is approximately 40 μL, these optimal in situ gels (P_13_H_2.5_-STF, P_13_H_2.5_HA-L_0.3_-STF, and P_13_H_2.5_HA-H_0.3_-STF) diluted with STF at a ratio of 40:7 (v/v) were used to mimic physiological situations upon administration.

#### 3.3.1. Temperature ramp

**Figure 3.**
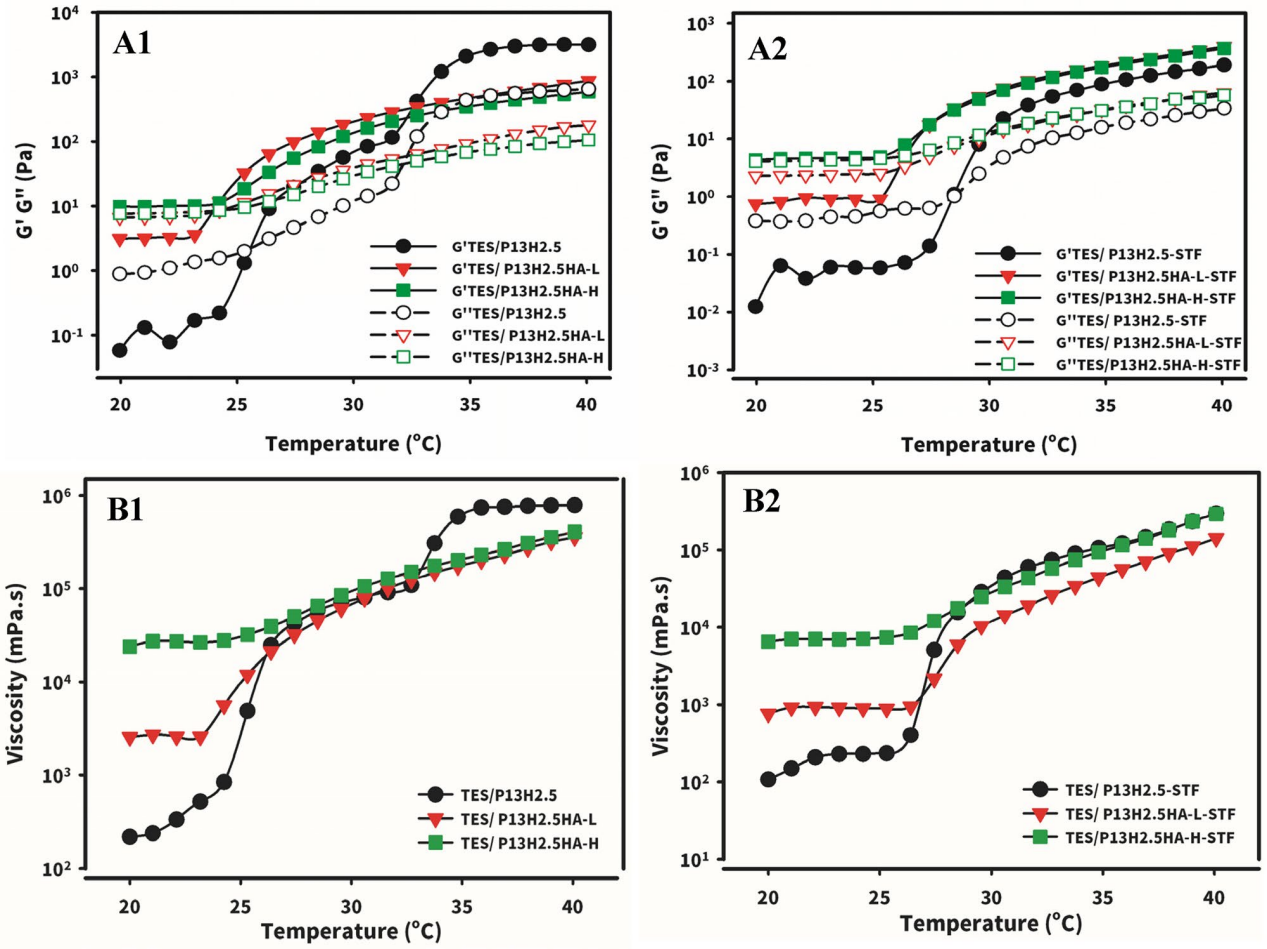
Temperature-viscoelasticity (A1&A2) and temperature-viscosity (B1&B2) ramp to investigate the viscoelasticity and the apparent viscosity as a function of the temperature for TES/HPβCD in situ gels based on poloxamer (13%) and HPMC (2.5%) with addition of either HA-L or HA-H at 0.3% without (A1&B1) or with (A2&B2) dilution at 40:7 (v/v) with STF.

Both viscoelasticity and apparent viscosity as a function of temperature for the optimized formulations were measured without (TES/P_13_H_2.5_, TES/P_13_H_2.5_HA-L_0.3_, and TES/P_13_H_2.5_HA-H_0.3_) or with (TES/P_13_H_2.5_-STF, TES/P_13_H_2.5_HA-L_0.3_-STF, and TES/P_13_H_2.5_HA-H_0.3_-STF) dilution with STF (Figure 3(A) and (B)), respectively. As presented in Figure 3(A1) and (A2) showing G′ and G′′ as a function of temperature, the T_sol-gel_ of TES/P_13_H_2.5_, TES/P_13_H_2.5_HA-L_0.3_, and TES/P_13_H_2.5_HA-H_0.3_ was determined to be 26.7 °C ± 1.4 °C, 24.1 °C ± 0.5 °C, and 23.5 °C ± 0.6 °C, respectively, and the T_sol-gel_ of those diluted with STF (TES/P_13_H_2.5_-STF, TES/P_13_H_2.5_HA-L_0.3_-STF, and TES/P_13_H_2.5_HA-H_0.3_-STF) was determined to be 28.8 °C ± 0.6 °C, 26.7 °C ± 1.3 °C, and 26.3 °C ± 1.8 °C, respectively. Irrespective of its MW, HA reduced T_sol-gel_, and a great extent of decrease was noted in the formulations containing 0.3% HA-H. The T_sol-gel_ of the three optimized formulations diluted with STF increased compared with that of their respective formulations without STF dilution. The three optimized formulations with or without STF dilution were maintained at a sol state (G′′ > G′) before instillation at a temperature of 20 °C–25 °C. After instillation, those three optimized formulations at a sol state were evenly spread over the ocular surface and then underwent sol–gel transition at a precorneal temperature of 34 °C, eventually converting into a hard gel (G′ > G′′) to remain for a longer time on the ocular surface for delivering testosterone loaded in the respective formulations.

The apparent viscosity measured as a function of temperature with a shear rate of 0.03 s ^− 1^ (a lower destructive rate during interblinking) for those optimized formulations without (TES/P_13_H_2.5_, TES/P_13_H_2.5_HA-L_0.3_, and TES/P_13_H_2.5_HA-H_0.3_) or with (TES/P_13_H_2.5_-STF, TES/P_13_H_2.5_HA-L_0.3_-STF, and TES/P_13_H_2.5_HA-H_0.3_-STF) STF dilution is presented in Figure 3(B1) and (B2), respectively. As shown in Figure 3(B1), the apparent viscosity of TES/P_13_H_2.5_ steadily increased with increasing temperature before reaching its T_sol-gel_ and then markedly increased with increasing temperature when the temperature reached close to its T_sol-gel_, followed by plateauing at a precorneal temperature of 34 °C. The apparent viscosity of TES/P_13_H_2.5_HA-L_0.3_ and TES/P_13_H_2.5_HA-H_0.3_ steadily increased at a lower extent with increasing temperature before reaching their T_sol-gel_ but only smoothly increased with increasing temperature when the temperature reached their T_sol-gel_ and then plateaued at a precorneal temperature of 34 °C. As illustrated in Figure 3(B1), the apparent viscosity at a room temperature of 25 °C for the three hydrogel formulations examined was in the order of TES/P_13_H_2.5_ < TES/P_13_H_2.5_HA-L_0.3_ < TES/P_13_H_2.5_HA-H_0.3_, whereas the order of the apparent viscosity after conversion into the gel state at a precorneal temperature of 34 °C was in the order of TES/P_13_H_2.5_ > TES/P_13_H_2.5_HA-L_0.3_ ≅ TES/P_13_H_2.5_HA-H_0.3_. Both TES/P_13_H_2.5_HA-L_0.3_ and TES/P_13_H_2.5_HA-H_0.3_ formed a higher viscous gel than did TES/P_13_H_2.5_ at room temperature, whereas TES/P_13_H_2.5_ formed a harder gel than did both TES/P_13_H_2.5_HA-L_0.3_ and TES/P_13_H_2.5_HA-H_0.3_ after transition into a gel state at a precorneal temperature of 34 °C. As illustrated in Figure 3(B2), the apparent viscosity measured with respect to temperature for the three optimized formulations with STF dilution followed the similar pattern as that observed for those without STF dilution with less viscosity than that of each of the corresponding formulations.

Before instillation, the apparent viscosity of formulations at room temperature should be lower to minimize imprecision in the volume of eye drops used for instillation and enable eye drops to evenly spread over the ocular surface after instillation. After instillation, the viscosity of formulations at a precorneal temperature of 34 °C should be markedly increased to its maximum to prevent its drainage because less viscous liquid enhances precorneal residence time. The apparent viscosity versus temperature profiles are illustrated in Figure 3(B1) and (B2). In summary, the three optimized formulations without or with STF dilution maintained a liquid state with less viscosity before instillation at a room temperature of 20 °C–25 °C. After instillation, they were evenly spread over the ocular surface and then underwent sol–gel transition at a precorneal temperature of 34 °C, eventually converting into a gel state, which enabled the three optimal formulations to remain longer on the ocular surface for delivering testosterone loaded in the respective formulations.

#### 3.3.2. Steady shear deformation

**Figure 4.**
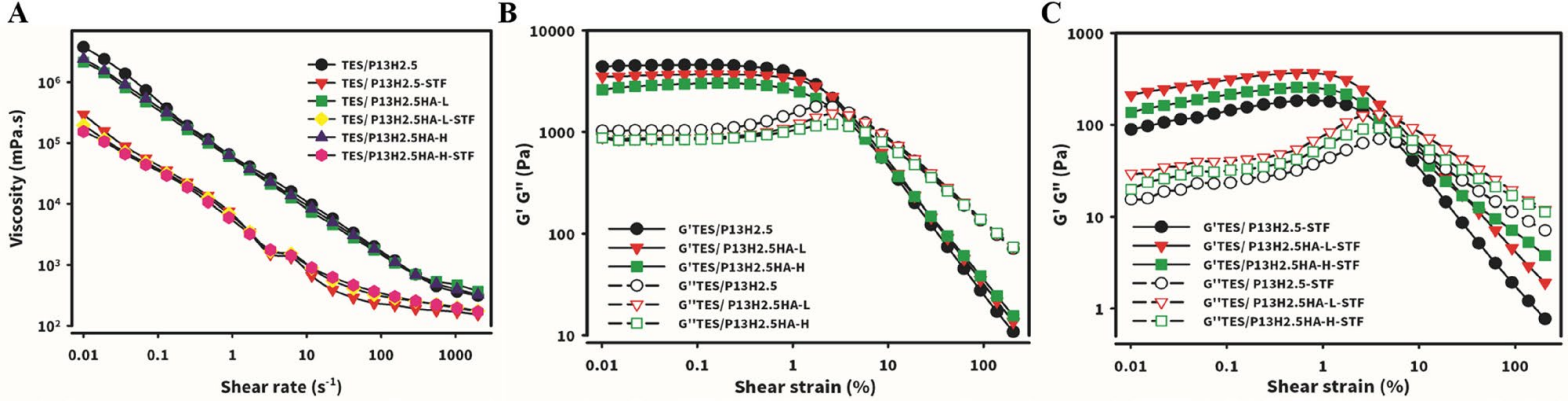
Steady shear deformation and Oscillatory deformation (amplitude sweep): (A) Apparent viscosity as a function of shear rate for Tes/HPβCD in situ gels based on poloxamer (13%) and HPMC (2.5%) with addition of either HA-L or HA-H at 0.3% concentrations without or with dilution at 40:7 (v/v) with STF. Elastic (full symbols, G’) and viscous (empty symbols, G”) moduli as a function of shear strain (0.01%-200%) for Tes/HPβCD in situ gels based on poloxamer (13%) and HPMC (2.5%) with addition of either HA-L or HA-H at 0.3% concentrations without (B) or with (C) dilution at 40:7 v/v with STF. Angular frequency 10 rad/s. Measurements were performed at 34 ◦C.

The steady shear deformation of the three in situ gel formulations was examined at a shear rate ranging from 0.01 to 2000s ^− 1^ and a precorneal temperature of 34 °C, and the results are presented in Figure 4(A). All the three in situ gel formulations (TES/P_13_H_2.5_, TES/P_13_H_2.5_HA-L_0.3_, and TES/P_13_H_2.5_HA-H_0.3_) behaved as shear-thinning fluids and exhibited non-Newtonian pseudoplastic flow behavior without or with STF dilution (40:7 v/v ratio). We observed a high viscosity at a low shear rate; this improved the tolerability of the hydrogels. Because the ocular shear rate varies from 0.03 s ^− 1^ (during interblinking) to 4500–28.0 s ^− 1^ (during blinking), the pseudoplastic flow behavior of the three optimized in situ hydrogels can be beneficial to the ocular drug delivery system. A study reported that the tear film exhibited pseudoplastic behavior and the application of an eye drop should not affect this property (Kesavan et al., 2010). Second, the shear rate during blinking was high (approximately 4500–28.0 s ^− 1^). The shear-thinning fluids exhibited low viscosity and enabled eye drops to spread smoothly over the ocular surface after administration. In addition, a considerably high viscosity at a high shear rate would cause difficulty on blinking. Finally, the formulation acted as a high-viscosity fluid at an ocular shear rate at 0.03 s ^− 1^ during interblinking. High-viscosity fluids reduce the drainage rate and improving precorneal residence time (Zhu & Chauhan, 2008). In summary, the pseudoplastic behavior of these samples improved the tolerability of drug drainage during the interblinking period and did not cause irritation due to shear thinning during the eye blinking.

#### 3.3.3. Oscillatory deformation (amplitude sweep)

The elastic G′ and viscous G′′ moduli of the three in situ gels (TES/P_13_H_2.5_, TES/P_13_H_2.5_HA-L_0.3_, and TES/P_13_H_2.5_HA-H_0.3_) were measured as a function of the strain amplitude γ_A_, and the results are illustrated in Figure 4(B) and (C), respectively, before and after STF dilution. The results revealed that the formed hard gel had a type III response, indicating a decrease in G′ and an increase followed by a decrease in G′′ (Hyun et al., 2002; Gugleva et al. 2020). The type III response is considered as the typical response of P407 hard gels (Li et al., 2018; Li & Hyun, 2018). At a low value of γ_A_, known as the plateau region, both G′ and G′′ moduli did not change markedly with increasing γ_A_. At a definite value of γ_A_, G′ decreased progressively, whereas G′′ reached the maximum. All the three optimal formulations had a higher elastic modulus than the viscous modulus at a low strain amplitude. Because of the rupture of the lattice microstructure, G′′ increased, and the further decreases in G′ and G′′ might result from the alignment of the layer and slide in the flow direction (Hyun et al, 2006). Favorable type III behavior expressed by these three hydrogel formulations indicated that the active ingredient during the application is homogeneously spread, which was attributed to the decrease in elasticity at a high amplitude of deformation. In summary, this characteristic is related to the alignment of layers in the flow direction (Hyun et al., 2006) and prevented the formation of large clusters that can lead to the uneven spread of the active substance often observed in soft gels exhibiting type IV behavior (Gugleva et al. 2020).

#### 3.3.4. Oscillatory deformation (frequency sweep)

**Figure 5.**
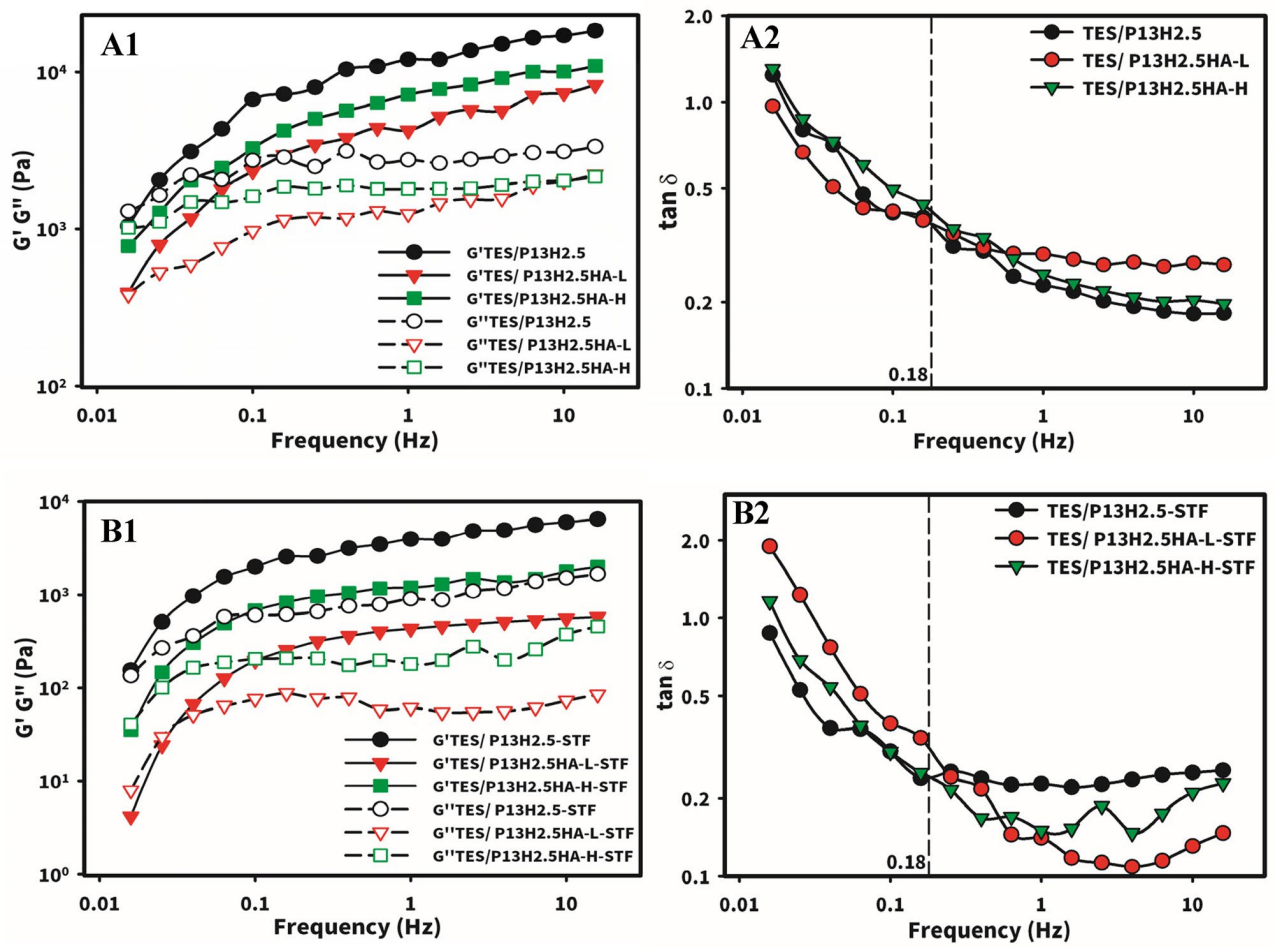
Oscillatory deformation (frequency sweep): Elastic (full symbols) and viscous (empty symbols) moduli (A1 and B1) and tanδ (A2 and B2) as a function of frequency of oscillations (0.1–100 rad/s) for TES/HPβCD in situ gels based on poloxamer (13%) and HPMC (2.5%) with the addition of either 0.3% HA-L or HA-H without (A1 and A2) or with (B1 and B2) STF dilution at a ratio of 40:7. Shear strain = 0.1% and T = 34 °C.

To analyze the viscoelastic properties of the formed gels (measured temperature of 34 °C was above T_sol-gel_) under small deformations (0.1%), we performed a frequency sweep test, and the results are presented in Figure 5(A) and (B) for the three hydrogel formulations without or with STF dilution, respectively. As shown in Figure 5(A1), the three optimized formulations consistently behaved as a hard hydrogel, with their G′ being considerably higher than G′′ at a higher angular frequency. By contrast, these formulations behaved as a soft hydrogel, with their G′ gradually approaching the corresponding G′′ at a lower angular frequency and an even lower G′′ at the lowest angular frequency. The same tendency was maintained even after dilution with STF (Figure 5(B1)). For viscoelastic materials (VEMs) such as hydrogels, oscillation deformation applied at a higher angular frequency led to inadequate time for energy dissipation, resulting in VEMs behaving more like solids with their G′ being higher than G′′. By contrast, oscillation deformation applied at a lower angular frequency led to sufficient time for energy dissipation, resulting in VEMs behaving more like liquids, with their G′ being gradually close to G′′ and even lower than viscous modulus G′′ with a decreasing angular frequency.

On average, most individuals blink approximately 9 to 13 times each minute, and each blink lasts between 0.1 and 0.4 s; this can be translated into an average oscillation deformation frequency of 0.18 s ^− 1^ due to eye blinking (Wang et al., 2011). The plot of tan δ (defined as the ratio of G′′ to G′) versus frequency was constructed for the three hydrogel formulations (TES/P_13_H_2.5_, TES/P_13_H_2.5_HA-L_0.3_, and TES/P_13_H_2.5_HA-H_0.3_) without or with STF dilution, respectively (Figure 5(A2) and (B2)). The value of tan δ at the average applied frequency of 0.18 s ^− 1^ was 0.371 and 0.241, 0.380 and 0.313, and 0.421 and 0.244, respectively, for the three hydrogel formulations without or with STF dilution. The tan δ value indicates the overall behavior of the hydrogel (Destruel et al., 2020). If the tan δ value is <1, the hydrogel is more elastic and similar to a block of gelatin. If the tan δ value is >1, the hydrogel is similar to honey in viscosity. Because thermosensitive hydrogels at above sol–gel transition temperature are in a solid instead of a liquid state, their tan δ values are usually <1, with thicker hydrogels having a lower tan δ value than do thin hydrogels. Thus, the tan δ value may be useful in predicting the cohesivity of hydrogels. The cohesivity of hydrogels is defined as the ability of hydrogels to deform without breaking or its shear malleability because of the affinity of its molecules to each other. Hydrogels with a higher tan δ value behave similar to warm butter spread on toasts; they dissipate more energy readily because they are more viscous and thus more easily glide or flow along the corneal surface. Because of this deformability, they can tolerate a higher amount of shear stress without breaking. By contrast, hydrogels with a lower tan δ value are more rigid and behave similar to refrigerated butter on toast, fragmenting more easily due to shear stress. Therefore, the cohesivity of hydrogels appears to be crucial in determining their even spread, allowing hydrogels to smoothly adapt to the ocular surface (Almeida et al., 2013). A comparison of the tan δ value for oscillation deformation due to eye blinking applied at frequencies ranging from 0.25 to 0.33 s ^− 1^ for the three hydrogel formulations without or with STF dilution demonstrated that the cohesivity followed the order of TES/P_13_H_2.5_HA-H_0.3_ > TES/P_13_H_2.5_HA-L_0.3_ > TES/P_13_H_2.5_ for those without STF dilution and TES/P_13_H_2.5_HA-L_0.3_ > TES/P_13_H_2.5_HA-H_0.3_ > TES/P_13_H_2.5_ for those with STF dilution. In summary, these results indicate that despite the addition of HA with different MWs, the hydrogel formulations with or without STF dilution increase the cohesivity by increasing the viscous behavior of hydrogels without the excessive deterioration of elastic behavior, facilitating the even spreading of the hydrogel formulations on the corneal surface at a normal blinking frequency and potentially resulting in longer retention time for enhanced drug delivery.

#### 3.3.5. Gel resistance under simulated eye blinking

To investigate the rheological behavior of the three optimized hydrogel formulations under simulated eye blinking, we performed two rheological experiments under the conditions of simulated eye blinking and interblinking, as described previously (Destruel et al., 2020). The formulations were subjected to 10 cycles of simulated eye blinking (i.e. a high shear rate interposed by interblinking periods with a low shear rate) to evaluate rheological behavior over time on the ocular surface.

##### 3.3.5.1. Rotational measurements

**Figure 6.**
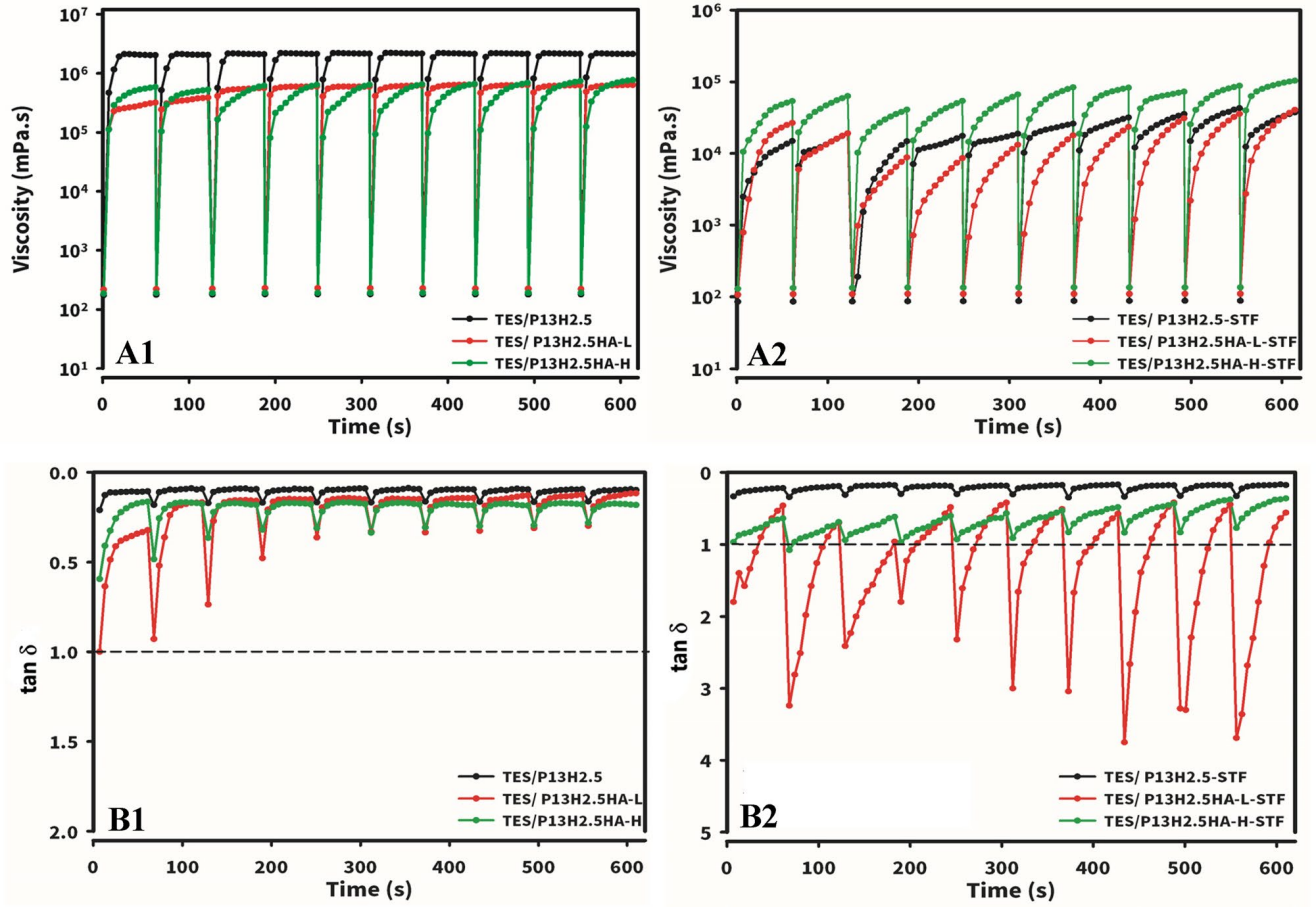
Viscosity (A1 and A2) and tanδ (B1 and B2) under simulated eye blinking for TES/HPβCD in situ gels based on poloxamer (13%) and HPMC (2.5%) with the addition of either 0.3% HA-L or 0.3% HA-H without (A1 and B1) or with (A2 and B2) STF dilution at a ratio of 40:7 by exposing alternatively to a high shear rate of 5000 s ^− 1^ for 1 s, immediately followed by measurements at a low destructive shear rate of 0.03 s ^− 1^ for 1 min for the determination of viscosity or immediately followed by measurements at a nondestructive shear rate for 1 min at an frequency of 1.0 Hz and an amplitude of 0.1% for the determination of tanδ at 34 °C.

We measured the viscosity of the three optimized hydrogel formulations without (TES/P_13_H_2.5_, TES/P_13_H_2.5_HA-L_0.3_, and TES/P_13_H_2.5_HA-H_0.3_) or with (TES/P_13_H_2.5_-STF, TES/P_13_H_2.5_HA-L_0.3_-STF, and TES/P_13_H_2.5_HA-H_0.3_-STF) STF dilution during interblinking intervals. At each simulated blinking at a higher shear rate of 5000s ^− 1^, the viscosity reached the minimum due to shear-thinning behavior and then increased during interblinking (Figure 6(A1) and (A2)) for the three optimized hydrogel formulations without or with STF dilution, respectively. As illustrated in Figure 6(A1), the viscosity of the three optimized formulations without STF dilution reached a plateau at its initial value almost immediately during interblinking periods accompanied by a gradual decline in viscosity all along the 10 cycles of simulated blinking. The order of the magnitude of viscosity at the plateau measured at 0.03 s^− 1^ was similar to that previously observed: TES/P_13_H_2.5_ > TES/P_13_H_2.5_HA-L_0.3_ and ≅ TES/P_13_H_2.5_HA-H_0.3_. The viscosity of the three optimized formulations diluted with STF (Figure 6(A2)) partially reached at its original value, followed by a gradual increase in viscosity to its maximum during interblinking periods all along the 10 cycles of simulated blinking. However, the order of magnitude of viscosity at their maximum measured at 0.03 s^− 1^ was TES/P_13_H_2.5_-STF ≅ TES/P_13_H_2.5_HA-L_0.3_-STF ≪ TES/P_13_H_2.5_HA-H_0.3_-STF. These results indicate that the viscosity of the three optimized hydrogel formulations attained a plateau at its initial value immediately during rest periods, exhibiting a steady viscosity all along the 10 cycles of simulated blinking. By contrast, although the viscosity of the three optimized hydrogel formulations recovered gradually, reaching their maximum with lower viscosity than that of the formulations without STF dilution during interblinking intervals, the delay appeared to not cause the loss of viscosity during interblinking, resulting in a steady viscosity all along the 10 cycles of simulated blinking. In summary, the three optimized hydrogel formulations, irrespective of STF dilution, maintained their nonthixotropic properties, thus evenly spreading and retaining the formulations on the ocular surface for extended ocular delivery.

##### 3.3.5.2. Oscillatory measurements

**Figure 7.**
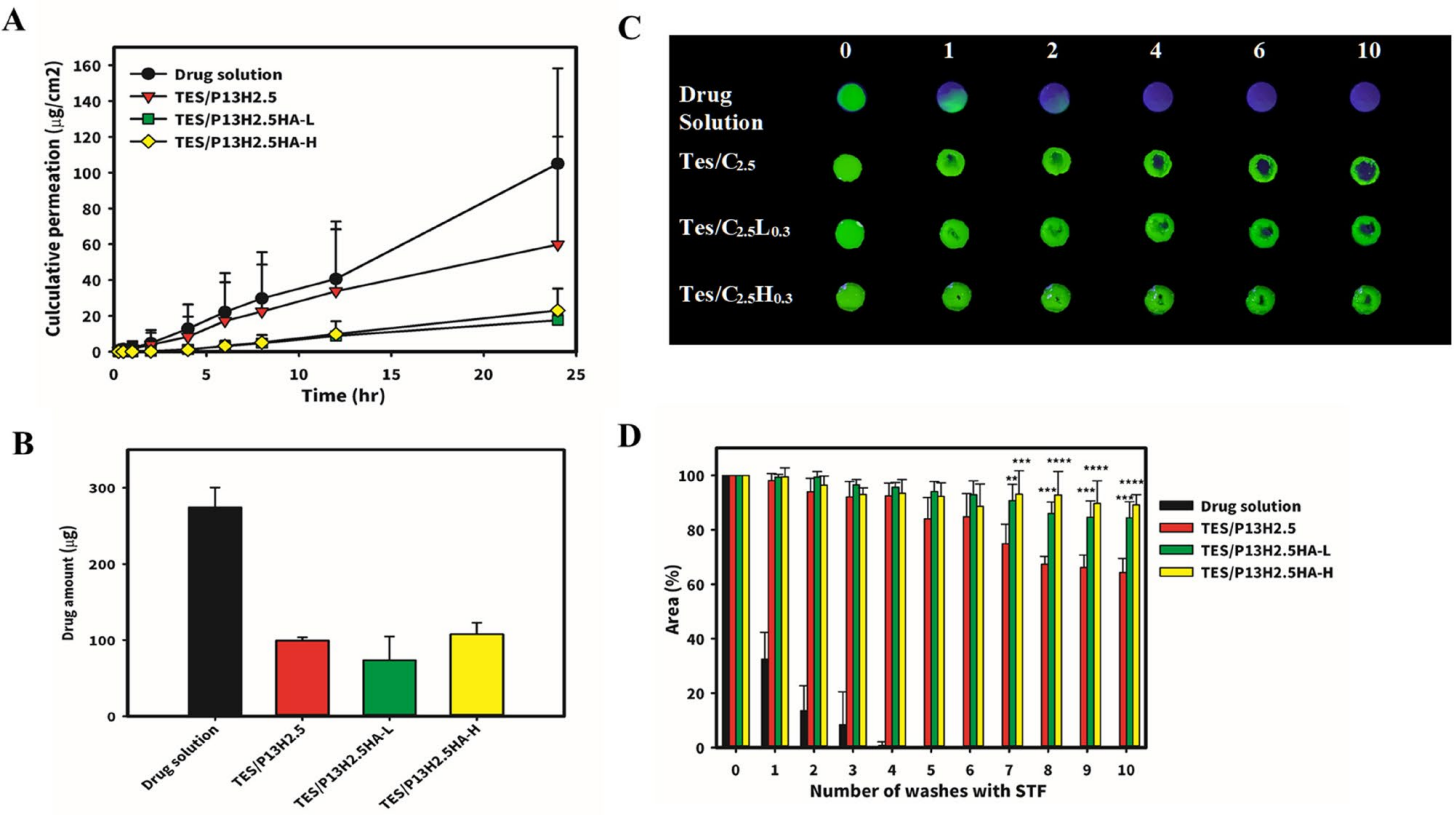
Ex vivo permeantion studies and in vitro retention studies of P407 formulations. Permeation of the different types of in situ gels through the excised rabbits’ cornea (A) and drug amount remained extrating by excised rabbits’ cornea after transcorneal permeation (B). (C) The fluorescence images and (D) the quantified fluorescence area of retention of P407 formulations on glass surface after a number of consecutive washes (200 mL/wash) using simulated tear fluid (STF). (** represents a significant difference at *p* < .01, *** represents a significant difference at *p* < .005, **** represents a significant difference at *p* < .001 compared to the area of residence formulation of Tes/C2.5).

We measured G′ and G′ moduli during interblinking intervals. The factor tan δ was used to examine the gel state of the formulations. At each simulated eye blinking, tan δ reached the maximum due to the partial deconstruction of the tridimensional network of the gel and then recovered during interblinking periods (Figure 7(B1) and (B2)). All the three optimized hydrogel formulations without STF dilution exhibited a tan δ value of <1 all along the 10 cycles. This result indicated that the gel network resisted the high shear of simulated blinking and remained at a gel state. Therefore, compared with the drug solution, the gel network can be retained on the ocular surface during blinking. However, among the three optimized hydrogel formulations diluted with STF, TES/P_13_H_2.5_-STF displayed a tan δ value of <0.5 all along the 10 cycles, remaining at a gel state, and TES/P_13_H_2.5_HA-H_0.3_-STF displayed a tan δ value close to 1.0 at a high shear rate and gradually exhibited a tan δ value of <1.0 all along the 10 cycles. Therefore, TES/P_13_H_2.5_HA-H_0.3_-STF could be partly cleared from the ocular surface as a liquid at the time of blinking. By contrast, TES/P_13_H_2.5_HA-L_0.3_-STF presented a tan δ value of >1.0, except at the moment before being subjected to a high shear rate of blinking. Subsequently, the gel network was destroyed at a tan δ value of >1, and the formulation converted to a liquid state. Hence, after instillation into the eye, the elimination rate of TES/P_13_H_2.5_HA-H_0.3_-STF from the ocular surface might be slightly higher than that of TES/P_13_H_2.5_-STF. Therefore, TES/P_13_H_2.5_HA-H_0.3_-STF exhibited more suitable rheological properties compared with TES/P_13_H_2.5_HA-L_0.3_-STF in terms of retaining a higher amount of hydrogel on the ocular surface for a longer time.

### 3.4. Ex vivo transcorneal permeation study

We examined the transcorneal permeation of testosterone by using an ex vivo rabbit cornea model, and the results are presented in Figure 7(A). A commercial eye drop containing testosterone for the treatment of DED is not yet available; thus, we used 0.5% testosterone with 10% HPβCD in distilled water as a control (drug solution). Passive diffusion of drugs across the cornea is affected by solubility, partition coefficient, MW, and degree of ionization (Almeida et al., 2013). In particular, the lipophilicity of a penetrant is among the most crucial factors for penetration because the top two layers of the cornea act as a barrier against hydrophilic drugs. Testosterone exhibits a high lipophilicity property. Therefore, with improved solubility, the transcorneal penetration ability would increase. Permeability values were significantly higher in drug solution than in different types of P407 in situ gels (TES/P_13_H_2.5_, TES/P_13_H_2.5_HA-L_0.3_, and TES/P_13_H_2.5_HA-H_0.3_). The results could be explained by the polymeric gel-like structure affecting drug diffusion negatively. The drug residence time will be increased on the ocular surface due to increased viscosity and mucoadhesion. Moreover, the better permeation ability of drug solution enables the presence of a higher amount of testosterone in the cornea tissue (Figure 7(B)).

### 3.5. Ex vivo precorneal residence study

We examined the precorneal residence time of different formulations on glass surfaces and the freshly excised rabbit cornea (Al Khateb et al., 2016). The fluorescent images and the results of the quantified leftover area of the formulations under continuous washing by STF are presented in Figure 7(C) and (D), respectively. As illustrated in Figure 7(C), a glass slide was used as a nonmucosal surface control to determine whether any residence effect observed is related to the rheological properties of the samples instead of the mucoadhesive effect of the polymer and viscosity enhancer. Drug solution with 1% fluorescein sodium displayed a poor retention effect on the glass slide. Only 32% ± 10% of drug solution was left on the glass slide after a single wash, and drug solution was completely removed from the surface after four washes. By contrast, a stronger retention effect was observed for P407 in situ gels (TES/P_13_H_2.5_, TES/P_13_H_2.5_HA-L_0.3_, and TES/P_13_H_2.5_HA-H_0.3_). After 10 washes, >60% of all the formulations remained on the glass slide; this concentration significantly differed from that of drug solution (*P* < .001) due to the sol–gel phase transition of P407 in situ gels under experimental conditions. Among them, both TES/P_13_H_2.5_HA-L_0.3_ and TES/P_13_H_2.5_HA-H_0.3_ demonstrated a better retention effect than did TES/P_13_H_2.5_ after seven washes (*P* < .01 and *P* < .005, respectively). After 10 washes, 84% ± 6% and 89% ± 4% of TES/P_13_H_2.5_HA-L_0.3_ and TES/P_13_H_2.5_HA-H_0.3_, respectively, remained on the glass slide, indicating their stronger ability to withstand the effect of STF fluid washes. By contrast, approximately only 64% ± 5% of TES/P_13_H_2.5_ was left on the glass surface after 10 washes. This finding indicates that although viscosity decreased after the addition of HA in TES/P_13_H_2.5_HA-L_0.3_ and TES/P_13_H_2.5_HA-H_0.3_, compared with TES/P_13_H_2.5_, HA still showed excellent resistance to STF washing and remained on the glass surface.

This might be explained by entangled networks provided by the gel structure. The backbone of HA is entangled by the synergy of the chemical structure of the molecule, internal hydrogen bonds, and interactions with the solvent, which contribute to a twisting ribbon structure in the solution. Meanwhile, due to its polyelectrolyte property, HA behaves as a semi-rigid chain in water. However, in a saline environment, the charges are screened, and HA appears as an expanded random coil (Dodero et al., 2019). Thus, because of the saline environment of STF and the entangled networks of HA, the networks of TES/P_13_H_2.5_HA-L_0.3_ and TES/P_13_H_2.5_HA-H_0.3_ were bound tightly together and did not disperse after STF washes. Moreover, no significant difference was noted between the retention area of TES/P_13_H_2.5_HA-L_0.3_ and TES/P_13_H_2.5_HA-H_0.3_ in each wash.

**Figure 8.**
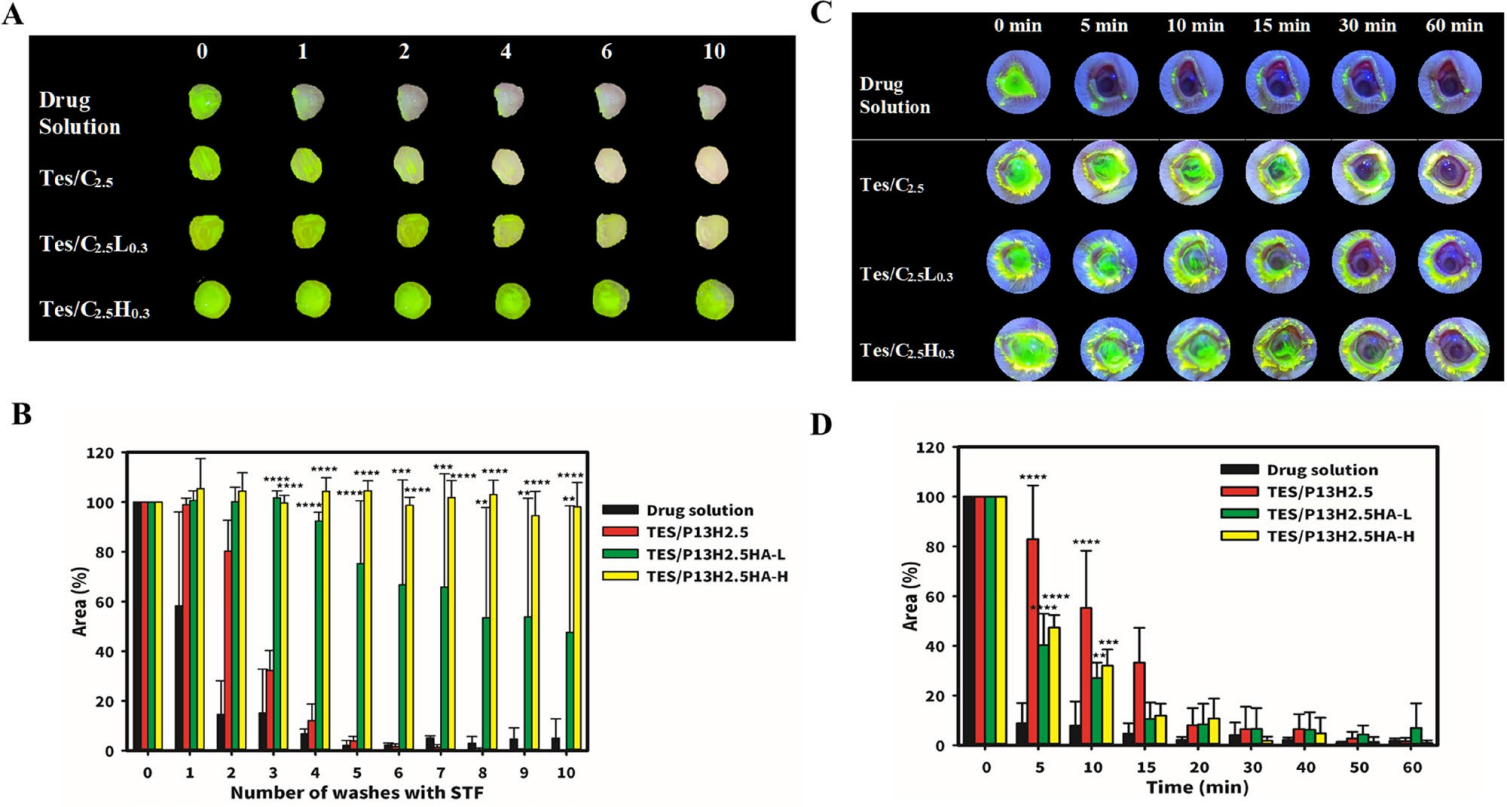
Ex vivo and in vivo retention studies of P407 formulations. (A) The fluorescence images and (B) the quantified fluorescence area of Retention of P407 formulations on cornea after a number of consecutive washes (200 mL/wash) using simulated tear fluid (STF). (** represents a significant difference at *p* < .01, *** represents a significant difference at *p* < .005, **** represents a significant difference at *p* < .001 compared to the area of residence formulation of TES/P_13_H_2.5_.) (C) The fluorescence images and (D) the quantified fluorescence area of retention of P407 formulations on the ocualr surface of living rabbits with in 60 minutes. (*** represents a significant difference at *p* < .01, *** represents a significant difference at *p* < .005, **** represents a significant difference at *p* < .001 compared to the area of residence formulation of drug solution.).

The results of the formulations applied on the excised rabbit cornea are presented as a plot of the fluorescein area versus number of washings in Figure 8(A) and the remaining percentage with respect to the number of washings in Figure 8(B). The drug solution demonstrated a similar result compared with the glass slide control group, with 30% ± 40% of drug solution remaining on the cornea after a single wash. By contrast, TES/P_13_H_2.5_ displayed a poorer retention effect on the cornea than the glass slide. This might be due to the attachment of normal saline that was used to store and maintain moisture in the cornea during transportation. Excessive water on the cornea affected the sol–gel transition by diluting the P407 concentration with STF, and this situation was more similar to the human cornea covered by the tear film. When the P407 concentration of the samples was decreased, TES/P_13_H_2.5_HA-L_0.3_ and TES/P_13_H_2.5_HA-H_0.3_ still exhibited an excellent retention effect. After four washes, approximately 92% ± 3% and 104% ± 5% of TES/P_13_H_2.5_HA-L_0.3_ and TES/P_13_H_2.5_HA-H_0.3_, respectively, remained on the cornea, compared with approximately 12% ± 7% of TES/P_13_H_2.5_ (*P* < .001). The high MW of HA appeared to prolong precorneal residence time compared with the low MW of HA. We noted that 98% ± 10% of TES/P_13_H_2.5_HA-H_0.3_ remained on the cornea compared with 48% ± 51% of TES/P_13_H_2.5_HA-L_0.3_ after 10 washes (*P* < .01).

The phenomenon can be explained on the basis of the findings of a previous study (Yadav et al., 2010). P407, HPMC, and HA are all mucoadhesive polymers that are well developed to provide intimate contact of the dosage form with the surface covered by mucin and to increase the residence time of the dosage form on the mucin layer for prolonging drug action. Although three of them displayed mucoadhesion property, HA exhibited the strongest mucoadhesive effect, which contributed to the longer residence time of the formulations containing HA, especially those with HA-H. In summary, reducing the P407 concentration in formulations might weaken the mucoadhesive effect of the formulations and the ocular bioavailability of testosterone. The addition of HA in dosage form can not only solve this problem by its excellent mucoadhesive effect but also alleviate DED symptoms due to the ability of HA to maintain ocular hydration. Because of the precorneal residence effect, the addition of HA-H into P407 in situ gels might be a potential dosage form for the ocular drug delivery system.

### 3.6. In vivo precorneal residence study

We performed an in vivo residence study on the ocular surface of New Zealand albino rabbits by using formulations (TES/P_13_H_2.5_, TES/P_13_H_2.5_HA-L_0.3_, and TES/P_13_H_2.5_HA-H_0.3_) containing fluorescein sodium. Each sample was applied in the rabbit’s right eye, whereas the left eye was treated as control. Data were collected in the form of the fluorescein area in photos analyzed by ImageJ. The fluorescein area versus time plots are presented in Figure 8(C), and the remaining percentage with respect to time was plotted in Figure 8(D). The drug solution rapidly washed out, with only 8.0% ± 8% left on the ocular surface after 5 min. In the same time interval, TES/P_13_H_2.5_HA-L_0.3_ and TES/P_13_H_2.5_HA-H_0.3_ exhibited a significantly longer residence time, with 40% ± 12% and 47% ± 5% left on the eyes compared with drug solution (*P* < .0001). TES/P_13_H_2.5_ displayed the best retention effect, with 82% ± 21% left on the eyes (*P* < .0001) compared with drug solution. After 15 min, the retention time of TES/P_13_H_2.5_, TES/P_13_H_2.5_HA-L_0.3_, and TES/P_13_H_2.5_HA-H_0.3_ still exhibited a higher retention effect than the drug solution (*P* < .01). Only TES/P_13_H_2.5_ significantly differed from the drug solution until 20 min. In summary, TES/P_13_H_2.5_ prolonged the precorneal residence time by four times compared with the drug solution, whereas TES/P_13_H_2.5_HA-L_0.3_ and TES/P_13_H_2.5_HA-H_0.3_ prolonged the precorneal residence time by three times compared with the drug solution.

The results of the in vivo residence study are not in agreement with those of the ex vivo residence study. This might be explained by the fact that the cornea for the in vivo residence study would be subject to the blinking of the eye, whereas no blinking of the excised cornea would occur in the ex vivo residence study. Rheological properties simulated under blinking conditions demonstrated that a hard gel still could be preserved with a tan δ value of <0.5 when TES/P_13_H_2.5_-STF was subjected to a high shear stress of simulated blinking. Thus, it could not be eliminated from the ocular surface as a liquid at the time of blinking. TES/P_13_H_2.5_HA-H_0.3_-STF displayed a tan δ value of ≤1.0 at a high shear rate and gradually decreased to a tan δ value of <1.0, enabling it to be eliminated from the ocular surface as a liquid at the time of blinking. By contrast, TES/P_13_H_2.5_HA-L_0.3_-STF presented a tan δ value of >1.0 except at the moment before being subjected to a high shear rate of blinking, indicating that TES/P_13_H_2.5_HA-L_0.3_-STF was maintained at a liquid state during the entire interblinking period. Therefore, the elimination rate from the ocular surface followed the order of TES/P_13_H_2.5_-STF < TES/P_13_H_2.5_HA-H_0.3_-STF ≅ TES/P_13_H_2.5_HA-L_0.3_-STF, which is similar to that observed in the in vivo residence study. This phenomenon could be attributed to the reduction in both P407 and HPMC concentrations in TES/P_13_H_2.5_HA-H_0.3_-STF and TES/P_13_H_2.5_HA-L_0.3_-STF by STF dilution after instillation, leading to conversion to a liquid state when subjected to a high shear rate at the time of blinking. Moreover, the addition of HA exerted an excellent mucoadhesive effect; however, it increased the distance between micelles with electrostatic repulsion and resulted in less micellar aggregation, leading to conversion into a gel state. This phenomenon affected the viscosity and gel-transforming rate of P407 in situ gels and weakened the retention effect of formulations when applied on the ocular surface of rabbits.

## 4. Conclusions

Poloxamer-based thermosensitive in situ hydrogels composed of 13% P407, 2.5% HPMC, and 0.3% HA were optimally screened using a low P407 concentration to minimize its potential side effects with enhancing mucoadhesion ability. Given the efficacy of testosterone in treating DED along with the lubricating effect of and enhancement of precorneal residence time by HA and HPMC, TES/P_13_H_2.5_HA-L_0.3_ and TES/P_13_H_2.5_HA-H_0.3_ could be used as an optimal ocular drug delivery system of HPβCD-solubilized testosterone. It was highly expectable that the ocular delivery of HPβCD-solubilized TES with P407-based thermogellable hydrogels could be prolonged to achieve effective clinical treatment of DED.

## Supplementary Material

Supplemental Material
