# Supplementary material for: Poloxamer sols endowed with in-situ gelability and mucoadhesion by adding hypromellose and hyaluronan for prolonging corneal retention and drug delivery
Source: Drug Deliv. 2023 Jan 1;30(1):2158964. doi: 10.1080/10717544.2022.2158964 (PMC9809414; doi:10.1080/10717544.2022.2158964)
Supplement: Supplemental Material [file IDRD_A_2158964_SM7733.docx]

**Supplementary Materials**


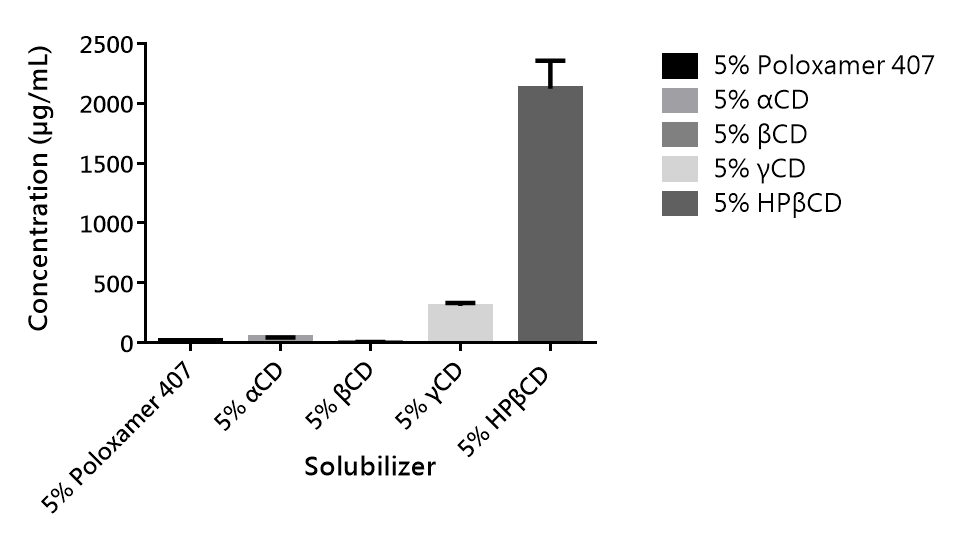


**Figure S1.** Saturation solubility of testosterone in distill water with 5% different types of solubilizers.

**(A)**

**(B)**


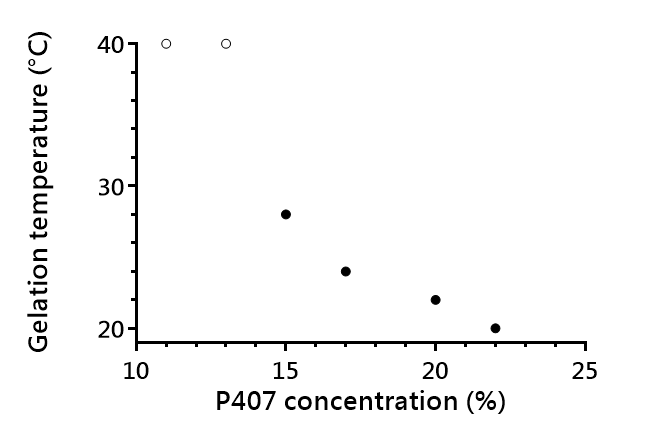


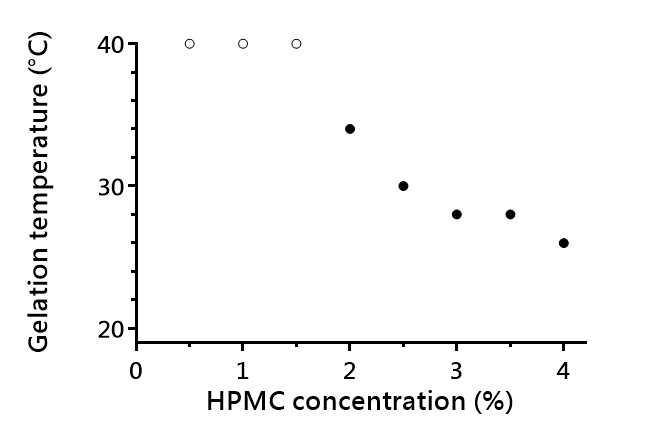


**(C)**


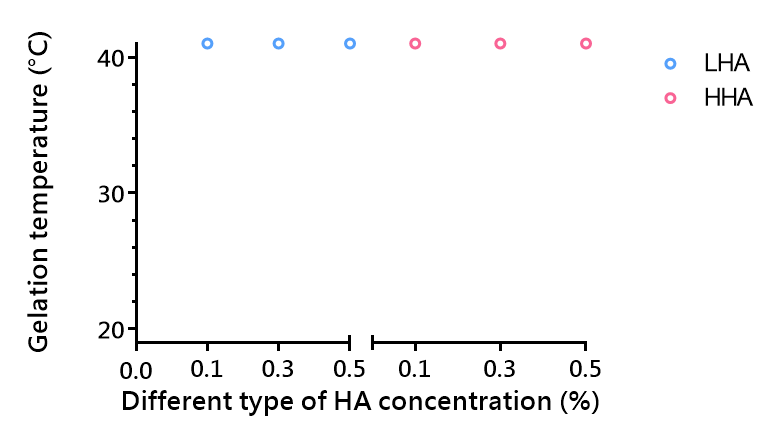


**Figure S2.** Effect of different concentration (11-22% w/w) of P407 (A) and 0.5-4.0% HPMC concentration (B), different concentration and molecular weight of HA (C) adding in 13% P407 gel on the sol-gel gelation temperature.。: no gelation effect in the range of temperature (4°C, 20-40°C).。: no gelation effect in the range of temperature (4°C, 20-40°C). HA-L: low molecular weight hyaluronic acid in this study (MW:700–900KDa). HA-H: high molecular weight hyaluronic acid in this study (MW:2000–2200KDa)
